# The Molybdenum Cofactor Biosynthesis Network: *In vivo* Protein-Protein Interactions of an Actin Associated Multi-Protein Complex

**DOI:** 10.3389/fpls.2017.01946

**Published:** 2017-11-14

**Authors:** David Kaufholdt, Christin-Kirsty Baillie, Rieke Meinen, Ralf R. Mendel, Robert Hänsch

**Affiliations:** Department of Plant Biology, Technische Universität Braunschweig, Braunschweig, Germany

**Keywords:** protein-protein interaction network, bimolecular fluorescent complementation (BiFC), split-luciferase, molybdenum cofactor, cytoskeleton, metabolic channelling

## Abstract

Survival of plants and nearly all organisms depends on the pterin based molybdenum cofactor (Moco) as well as its effective biosynthesis and insertion into apo-enzymes. To this end, both the central Moco biosynthesis enzymes are characterized and the conserved four-step reaction pathway for Moco biosynthesis is well-understood. However, protection mechanisms to prevent degradation during biosynthesis as well as transfer of the highly oxygen sensitive Moco and its intermediates are not fully enlightened. The formation of protein complexes involving transient protein-protein interactions is an efficient strategy for protected metabolic channelling of sensitive molecules. In this review, Moco biosynthesis and allocation network is presented and discussed. This network was intensively studied based on two *in vivo* interaction methods: bimolecular fluorescence complementation (BiFC) and split-luciferase. Whereas BiFC allows localisation of interacting partners, split-luciferase assay determines interaction strengths *in vivo*. Results demonstrate (i) interaction of Cnx2 and Cnx3 within the mitochondria and (ii) assembly of a biosynthesis complex including the cytosolic enzymes Cnx5, Cnx6, Cnx7, and Cnx1, which enables a protected transfer of intermediates. The whole complex is associated with actin filaments via Cnx1 as anchor protein. After biosynthesis, Moco needs to be handed over to the specific apo-enzymes. A potential pathway was discovered. Molybdenum-containing enzymes of the sulphite oxidase family interact directly with Cnx1. In contrast, the xanthine oxidoreductase family acquires Moco indirectly via a Moco binding protein (MoBP2) and Moco sulphurase ABA3. In summary, the uncovered interaction matrix enables an efficient transfer for intermediate and product protection via micro-compartmentation.

## Introduction

Molybdenum (Mo) belongs to the group of essential metals like iron, zinc, manganese, or copper, which are used as cofactors or in prosthetic groups. These metals ensure redox enzyme functions in all organisms (Hänsch and Mendel, 2009). The transition metal Mo is important due to its differing electron configurations (Holm et al., 2010). However, Mo is biologically inactive unless it is complexed by a specific prosthetic group. With the exception of the bacterial nitrogenase (Hu and Ribbe, 2013), all Mo-containing enzymes (Mo-enzyme) use Mo imbedded in a pterin based scaffold (Mendel, 2013). This molybdenum cofactor (Moco) is highly conserved during evolution and Moco containing enzymes can be found throughout all kingdoms of life (Schwarz and Mendel, 2006). Mo-enzymes are essential for the global carbon, sulphur, and nitrogen cycles (Hille, 2002).

**Figure 1:**
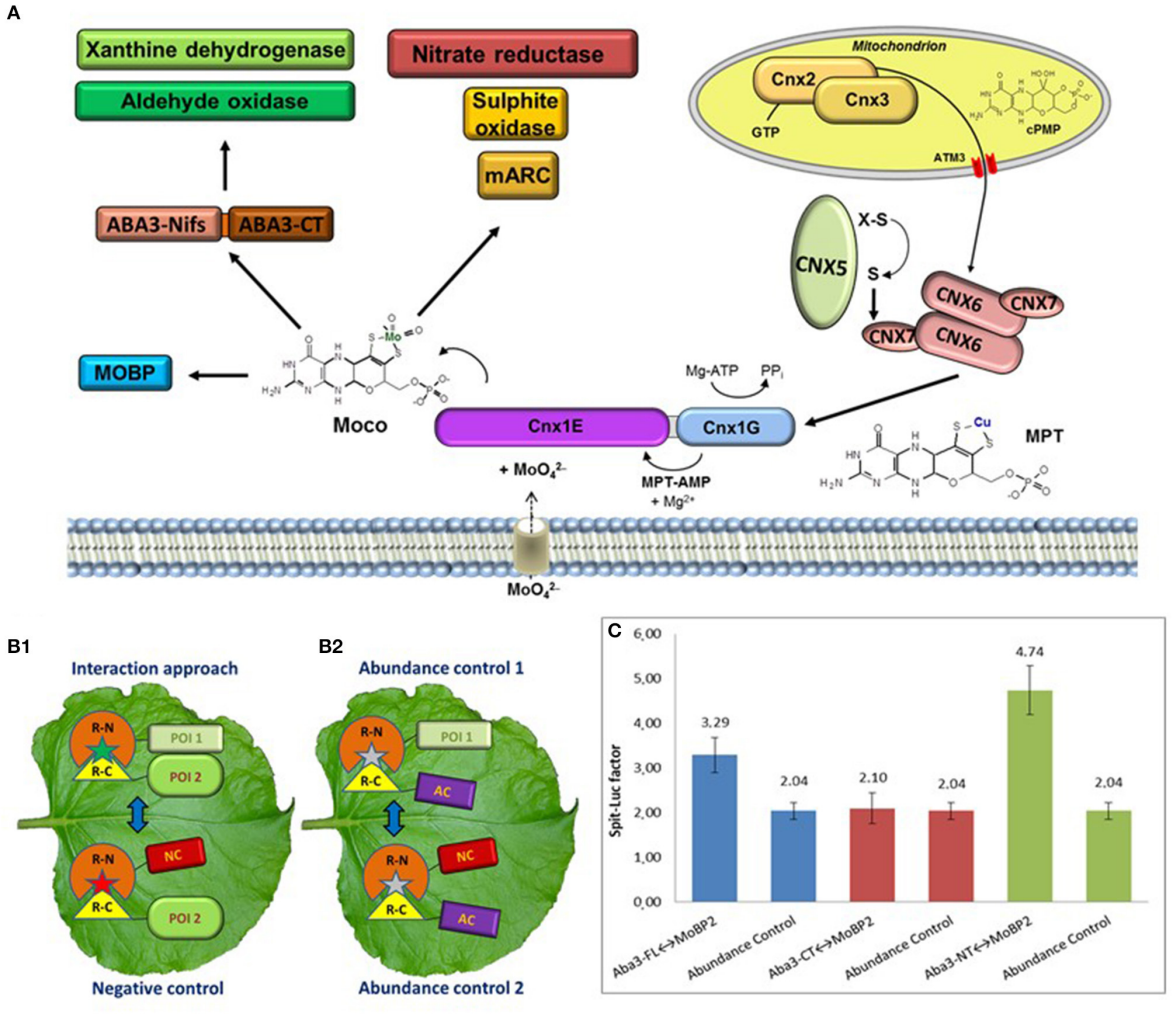
**(A)** The Moco enzyme pathway with the four-step reaction catalysed by Cnx2 and Cnx3 inside mitochondria as well as Cnx6, Cnx7, Cnx5, and Cnx1 in the cytosol. After biosynthesis, Moco is distributed to both families of Mo-enzymes and to Moco binding proteins. **(B)** Schematic presentation of an interaction approach and the necessary three controls for a split-protein assay. Depicted are the used constructs with the N-terminal (R-N) and the C-terminal (R-C) fragment of a reporter. **(B1)** The interaction approach needs two POI, while the negative control replace one POI for a non-interacting negative control protein (NC). **(B2)** In the abundance controls, a non-interacting abundance control protein (AC) is used. **(C)** FLuCI interaction study between ABA3 and MoBP2. Analysed was the full-length protein (FL) as well as the single N-terminal (NT) and C-terminal domain. Shown is the split-luciferase factor of the interaction approach in relation to the negative control. In addition, the split-luciferase factors of the two abundance controls are depicted, demonstrating the background factor without interaction. For interaction between the POI's, the split-luciferase factor has to be higher than the background factor.

The Moco biosynthesis four-step reaction pathway as well as the biosynthesis enzymes are highly conserved and can be found in nearly all prokaryotes and eukaryotes. These enzymes are designated in plants by an abbreviation for *cofactor for nitrate reductase and xanthine dehydrogenase* (Cnx1-3 and 5-7). As shown in Figure 1A, Cnx2 and Cnx3 catalyse the first step by converting 5'-GTP to cyclic pyranopterin monophosphate inside mitochondria (cPMP; Hänzelmann et al., 2002; Santamaria-Araujo et al., 2004). The resulting cPMP is exported into the cytosol by the ATM3 transporter (Teschner et al., 2010) for introducing a dithiolene group by transferring two sulphur atoms forming molybdopterin (MPT). This second reaction step is catalysed by the MPT synthase, a heterotetramer with two large Cnx6 and two small Cnx7 subunits. After sulphur transfer, the Cnx7 subunits need to be re-sulphurated via the MPT synthase sulphurase Cnx5 (Matthies et al., 2004). The Mo insertase Cnx1 catalyses the last two steps of biosynthesis. Cnx1 consists of a larger N-terminal Cnx1E domain and a smaller C-terminal Cnx1G domain. MPT is bound by the Cnx1G domain and is activated through adenylation, thus forming MPT-AMP in the third step (Kuper et al., 2003). This intermediate is transferred to the Cnx1E domain, which releases AMP and inserts Mo derived from molybdate (Llamas et al., 2006). This required molybdate is imported by specific molybdate transporters (Gasber et al., 2011; Tejada-Jiménez et al., 2013).

The five Mo-enzymes found in plants are classified in two families based on the coordination chemistry of Mo in their active centre (Mendel and Schwarz, 2011). The directly produced di-oxo form of Moco is used by the first family, named sulphite oxidase family (SO-family). The SO-family consists of three members: sulphite oxidase (SO; Hänsch and Mendel, 2005), nitrate reductase (NR; Campbell, 1999), and mitochondrial amidoxine reducing component (mARC; Havemeyer et al., 2006). The second Mo-enzyme family, the xanthine oxidoreductase family (XOR-family), consists of aldehyde oxidase (AO; Seo et al., 2000) and xanthine dehydrogenase (XDH; Hille and Nishino, 1995). The XOR-family possesses the sulphurised mono-oxo Moco, which is produced by an additional biosynthesis step through the Moco sulphurase ABA3 in Arabidopsis (Wollers et al., 2008) and FLACCA in tomato (Sagi et al., 2002).

For plant survival, the most important Mo-enzyme is the cytosolic NR. It catalyses the first step of nitrate assimilation. The conversion of nitrate to nitrite is essential for plant growth and development (Campbell, 1996). The peroxisomal SO enables stress tolerance against sulphur dioxide (Brychkova et al., 2015; Baillie et al., 2016). XDH has a role in the catabolism of purines by catalysing the oxidation of hypoxanthine to xanthine and finally to uric acid (Brychkova et al., 2008) while producing superoxide anions (Zarepour et al., 2010). AO is involved in the biosynthesis of the phytohormone abscisic acid (Seo et al., 2000) as well as in detoxification of carbonyl aldehydes in stressed Arabidopsis siliques (Srivastava et al., 2017). The dependence of plants on functional Mo-enzymes highlights the necessity of efficient and reliable Moco production as well as its correct insertion into the apo-enzymes. However, both Moco and its intermediates are described as highly oxygen sensitive (Rajagopalan and Johnson, 1992). Therefore, a freely diffusible pool of molecules in the cytosol is rather unlikely due to the high degradation risk during biosynthesis and transfer. This problem can be circumvented by a protection mechanism via direct transfer of intermediates from one protein to the next, which has not been described for any organism so far. However, such a metabolic channelling in a protein complex is an efficient strategy to protect sensitive molecules (Miles et al., 1999; James and Viola, 2002) which can be studied using molecular-biological methods on cellular level. The presented study on the Moco-biosynthesis complex can function as a blueprint for investigations of other physiological protein networks in plants.

## *In vivo* protein-protein interaction studies are powerful tools to analyse the plant interactome

A protected Moco transfer requires tight protein-protein interaction between involved proteins within the plant cell. *In vivo* protein-protein interaction studies were performed inside living cells thus providing natural conditions for protein-protein interaction. In contrast, *in vitro* experiments just give indications for the physiological processes but cannot mimic e.g., compartmentation and physiological micro-environment inside the living cell and thus lack the complexity of *in vivo* experiments. Conditions of *in vivo* experiments have to be monitored very accurately. The used concentration of proteins of interest (POI) in the cells depends on transformation and expression/degradation levels, respectively. Furthermore, results will be influenced by both reaction conditions as well as reaction partners depending on age, growing stage or vitality of the plant cells. These prerequisites have to be equalised by many experimental replications and the usage of appropriate negative and abundance controls. However, only *in vivo* interaction studies are able to verify the preconditions of a potential substrate channelling with so many partners in one protein complex.

The majority of developed *in vivo* interaction studies are based on fusion proteins consisting of the POI's and a split reporter protein (Stynen et al., 2012). There are only a few alternative *in vivo* methods like yeast-two-hybrid assay (Y2H; Causier and Davies, 2002) or Foerster resonance energy transfer (FRET; Bhat et al., 2006). The Y2H is often used for screenings for new interaction partners, however, the heterologous system counteract measuring in the natural environment. On the other hand, FRET studies are performed inside plant cells, but measurement and interpretation are much more sophisticated than for split reporter systems. Both the bimolecular fluorescence complementation (BiFC; Waadt et al., 2008; Gehl et al., 2009) and the floated leaf luciferase complementation imaging assay (FLuCI; Gehl et al., 2011), use the ability of a split reporter to reconstitute and to resume activity after bringing the split parts into close proximity. Interaction of the fused POI's increases the reconstitution of the reporter termini, which increases the measureable fluorescence or luminescence, respectively, of the analysed cells compared to a negative control with reconstitution only by chance (Figure 1B1). It has to be considered, that a fused reporter protein could mask an interaction site by steric hindrance. Therefore, all possible orientations of reporter fusions to both POI's have to be tested for reliable results.

All split-protein assays need additional controls to validate the results of the interaction approach (Figure 1B2). Random reconstitution depends on the concentrations of both fusion partners. However, the abundance of constructs with the POI and the negative control construct may be variable due to altered protein expression or degradation, which could yield false positive or negative results. Moreover, the behaviour of random reconstitution under *in vivo* conditions is multifaceted. This fact demanded the measurement of the background level of random reconstitution inside a living cell, called abundance control, which additionally has to be performed when measuring *in vivo* protein-protein interactions (Kaufholdt et al., 2013).

Both assays have differences in the nature of reconstitution of the used reporter proteins, which makes the combination of these assays reasonable. Fluorescence reporter termini in the BiFCs assay reconstitute irreversibly to full reporter functionality (Rose et al., 2010), which allows semi-quantitative measurement of interactions, even if these interactions are weak or only once occurring in a protein life cycle (Kaufholdt et al., 2016b). In contrast, the reconstituted luciferase termini of the FluCI assay are reversibly bound and able to disjoin from each other after the contact between the POI's is finished. This dynamic reporter system allows for the measurement of interaction strengths (Chen et al., 2008).

## Localisation of Moco pathway enzymes

The basis for a direct transfer of metabolites via protein-protein interaction is the localisation of all involved enzymes in the same cell compartment. Therefore, localisation of all Moco pathway proteins was investigated via fluorescence reporter studies. With exception of the enzymes of the first Moco biosynthesis step Cnx2 and Cnx3 that are localised inside mitochondria, the enzymes Cnx1, Cnx5, Cnx6, Cnx7, and ABA3 were all localised within the cytosol (Kaufholdt et al., 2013). The Mo-enzymes NR, XDH, and AO have also been localised within the cytosol (Dalling et al., 1972; Koiwai et al., 2004; Kaufholdt et al., 2016b). A tonoplast association is discussed for XDH (Ma et al., 2016). The exceptions are the peroxisomal SO (Nowak et al., 2004) and mARC, which was localised in the outer membrane of mitochondria in mammals (Havemeyer et al., 2006).

## Moco biosynthesis enzymes assemble into a multi-enzyme complex

Starting at reaction step one of the Moco biosynthesis, Kaufholdt et al. (2013) tested the mitochondrial protein pair Cnx2/Cnx3 using BiFC and FLuCI assays. Interaction approaches in organelles demand organelle-specific negative and abundance control proteins. Such a set of mitochondria-specific control vectors enabled interaction studies between the proteins of the first biosynthesis step Cnx2 and Cnx3. BiFC revealed an interaction between these two proteins and showed a punctual fluorescence distributed within the cytosol representing mitochondria. This interaction could be verified by the FLuCI assay.

Both Cnx2 and Cnx3 are unable to interact with the other Moco biosynthesis enzymes for direct cPMP transfer because of the localisation in different compartments (Figure 1). In addition, an interaction of these enzymes with the transporter ATM3 to transfer their product cPMP is still unknown. However, an unprotected export is also feasible because cPMP is the least oxygen sensitive Moco biosynthesis intermediate. Furthermore, cPMP is stable enough for cPMP-mediated therapy of human Moco deficiency, which is unique for Moco intermediates (Santamaria-Araujo et al., 2012).

As a next step, the cytosolic enzymes were analysed for interaction (Kaufholdt et al., 2013). MPT-synthase consists of two Cnx6 and two Cnx7 subunits. Interactions were found between the protein pairs Cnx6/Cnx6 and Cnx6/Cnx7 via BiFC and FLuCI assays. The interaction strength of both pairs is very high and the strongest of all protein pairs tested, which indicates a permanent contact of these proteins. Therefore, a stable heterotetrameric complex of the four subunits was concluded, which verified the results of the crystal structure analysis of *Escherichia coli* MPT-synthase complex by Rudolph et al. (2001).

The C-terminal region of the small MPT synthase subunit Cnx7 is highly conserved in all Moco depending organisms. The terminal double glycine motif carries the sulphur atom for transfer to cPMP and inserts into a pocket of each large subunit forming two probably independent active sites (Rudolph et al., 2001). Mutations of the glycine motif in *E. coli* (Schmitz et al., 2007) and human (Hänzelmann et al., 2002) homologs decreased the sulphur transfer activity dramatically. In the FLuCI-studies, Kaufholdt et al. (2013) investigated whether this functional impairment is caused by a disturbance of protein-protein interaction between the MPT subunits by point mutations of the penultimate glycine. The interaction strength of Cnx6 to wildtype and mutant Cnx7 were directly compared to each other. Both a mutation of glycine to a larger phenylalanine as well as to the charged glutamate decreased the interaction strength by a third and by half, respectively. This indicates that mutations in the last two glycines lead to a loss of function of the MPT-synthase due to a disturbance of the heterotetramer formation.

MPT-synthase sulphurase Cnx5 as well as the Moco insertase Cnx1 were tested each against both subunits of the MPT-synthase. Interactions were detected for the protein pairs Cnx5/Cnx7 and Cnx1/Cnx6, while the protein pair Cnx5/Cnx6 as well as Cnx1/Cnx7 showed no interaction. Therefore, a direct contact was concluded for the sulphur transfer from Cnx5 to Cnx7 as well as for the MPT transfer from Cnx6 to Cnx1. In addition, the E-domain of Cnx1 was identified as the interaction domain with Cnx6, which was also verified by additional crosslinking experiments (Kaufholdt et al., 2013). However, in contrast to the permanent interactions within the MPT-synthase, interaction strengths of the protein pairs Cnx5/Cnx7 and Cnx1/Cnx6 were distinct but less intensive, so they seem to be of transient nature.

## The Moco Biosynthesis Complex Interacts with the Actin Cytoskeleton via Cnx1 as Anchor Protein

Micro-compartmentation of biosynthesis complexes at the cytoskeleton is frequently observed in plant cells (Gutierrez et al., 2009; Marek et al., 2011). First indications for a cytoskeleton anchoring were also seen for the Moco biosynthesis complex. The plant Mo insertase Cnx1 has an animal homologue named gephyrin, which was shown to anchor neuronal postsynaptic inhibitory glycine receptors to polymerised tubulin (Kirsch et al., 1993). In addition, *in vitro* studies depicted that plant Cnx1 was co-sedimented with rabbit filamentous actin (Schwarz et al., 2000). To verify these indications inside the plant cell, BiFC assays were used by Kaufholdt et al. (2016a) for analyses of possible interactions of the Moco biosynthetic complex with both actin filaments and microtubules. A non-invasive indirect labelling of the cytoskeleton was used via cytoskeleton binding proteins fused to the reporter fragments. Direct fusion of cytoskeleton proteins disturbs cytoskeleton multimerisation or can mask binding sites. Two binding proteins were used for both cytoskeleton types: (i) Lifeact from *Saccharomyces cerevisiae* (Riedl et al., 2008; Era et al., 2009) and the actin binding domain 2 of fimbrin from *Arabidopsis thaliana* (Sano et al., 2005) for actin filaments as well as (ii) microtubule binding domains of the protein Casein-Kinase-1-Like-6 (Ben-Nissan et al., 2008) and the Microtubule Associated Protein 65-1 (Smertenko et al., 2004) from *A. thaliana* for microtubules.

Cnx1 was the only cytosolic Moco biosynthesis protein that showed an interaction with actin filaments. Beside a stronger fluorescence compared to the negative control, a specific nucleus-concentrated fluorescence pattern—forming a star-like pattern on the nuclear basket—also indicates interaction of the Cnx1 to the cytoskeleton in this kind of BiFC approach. A more detailed analysis of the two Cnx1 domains identified Cnx1G as the main interaction site of Cnx1 to filamentous actin. However, microtubule interaction was not detectable for any of the Moco proteins.

After identification of Cnx1 as an actin binding protein, anchoring of the entire Moco biosynthesis complex to actin filaments was hypothesized and therefore studied by a BiFC approach including labelled Cnx6 and actin binding proteins with fluorescence protein fragments. This approach with additionally expressed unlabelled Cnx1 formed the star-like pattern characteristic for an actin filament interaction. Therefore, Cnx1 acts as bridge protein binding to filamentous actin with its Cnx1G domain as well as to proteins of the Moco biosynthesis with its Cnx1E domain.

## Transient protein-protein interactions of Moco biosynthesis enzymes with Mo-enzymes allow a protected allocation of Moco

After biosynthesis, Moco has to be transferred to apo-Mo-enzymes in a protected way. Cnx1 delivers di-oxo Moco, which is prepared for incorporation into the apo-Mo-enzymes of the SO-family. BiFC assays indicate direct interaction in the cytosol of Cnx1 with NR and SO, respectively. This leads to the assumption of a Moco insertion before importing holo-SO into peroxisomes.

No interaction of Cnx1 neither with the mono-oxo Moco using Mo-enzymes XDH/AO nor with ABA3 was detectable, which excludes direct metabolite transfer. However, an indirect transfer is plausible, because the Moco binding protein MoBP2 might act as a potential bridge protein between Cnx1 and ABA3. An interaction with both enzymes was identified in a screening using the FLuCI assay. As example, the selected interaction study of ABA3 and MoBP2 is depicted in Figure 1C to demonstrate the necessity of abundance controls for identification of the N-terminal domain of ABA3 as exclusive interaction domain with MoBP2. In addition, a one-time-only interaction of XDH1 with the C-terminal domain of ABA3 was identified. Therefore, after processing of Moco a direct transfer from ABA3 to the apo-XOR-enzymes is suggested.

## Conclusion

*In vivo* protein-protein interaction assays helped to explain the multifaceted Moco biosynthesis protein network in subcellular compartments inside the plant cell. The irreversible complementation of the BiFC assay enabled to enlighten and to localise every type of interaction, permanent interactions of complexes as well as transient interactions. In contrast, the reversible and dynamic complementation of the luciferase reporter in the FLuCI assay enabled characterizing the interaction strength between protein pairs.

The presented data allow for generating an interaction matrix as well as defining interaction strengths for proteins of the Moco biosynthesis proteins in higher plants. This micro-compartmentation inside the cytosol is a precondition for metabolic channelling. During the passage through the three cytosolic reaction steps, the oxygen sensitive intermediates of Moco can be channelled from one protein to the next inside a biosynthesis complex. This protein complex structure permits an evolutionary physiological advantage by economising with the trace element molybdate via efficient resource and energy management.

Cnx1 anchors this whole complex on actin filaments as a bridge protein by mediating an indirect interaction of the MPT-synthase to the cytoskeleton. During sulphuration of Cnx7, Cnx5 is also part of this micro-compartmentation. Different hypotheses are possible to explain a spatial anchoring of Moco biosynthesis complex on the cytoskeleton for increasing biosynthesis efficiency. (i) Actin binding could increase the stability of the complex. Cnx1 first makes contact to actin followed by recruitment of the other components of the complex. A free diffusion in the cytosol could disturb these interactions and consequently the reaction efficiency. (ii) The anchoring could be important for positioning the whole complex near specific transporters to get the required substrates directly after import. One of these transporters is the mitochondrial ATM3 exporter, which transfers cPMP out of mitochondria. (iii) Another group of transporters provides molybdate for insertion into MPT by Cnx1. An efficient supply of molybdate is important for an uninterrupted reaction cascade inside the Moco biosynthesis complex. The animal Cnx1 homologue gephyrin clusters neuronal receptors at the postsynaptic membrane via microtubule binding. In an analogous manner, Cnx1 could cluster molybdate transporters on actin filaments for direct substrate channelling via protein interaction.

The interaction network between the Moco biosynthesis complex and the Mo-enzymes forms a potential allocation pathway reducing the risk of oxygen damage of Moco. Mo-enzymes using di-oxo Moco have a direct interaction with the biosynthesis complex while Mo-enzymes using mono-oxo Moco receive the prosthetic group via an indirect pathway. Furthermore, the bypass of Moco via a binding protein and ABA3 leads to the hypothesis that di-oxo Moco is processed prior to insertion into the members of the XOR-family.

**Figure 2:**
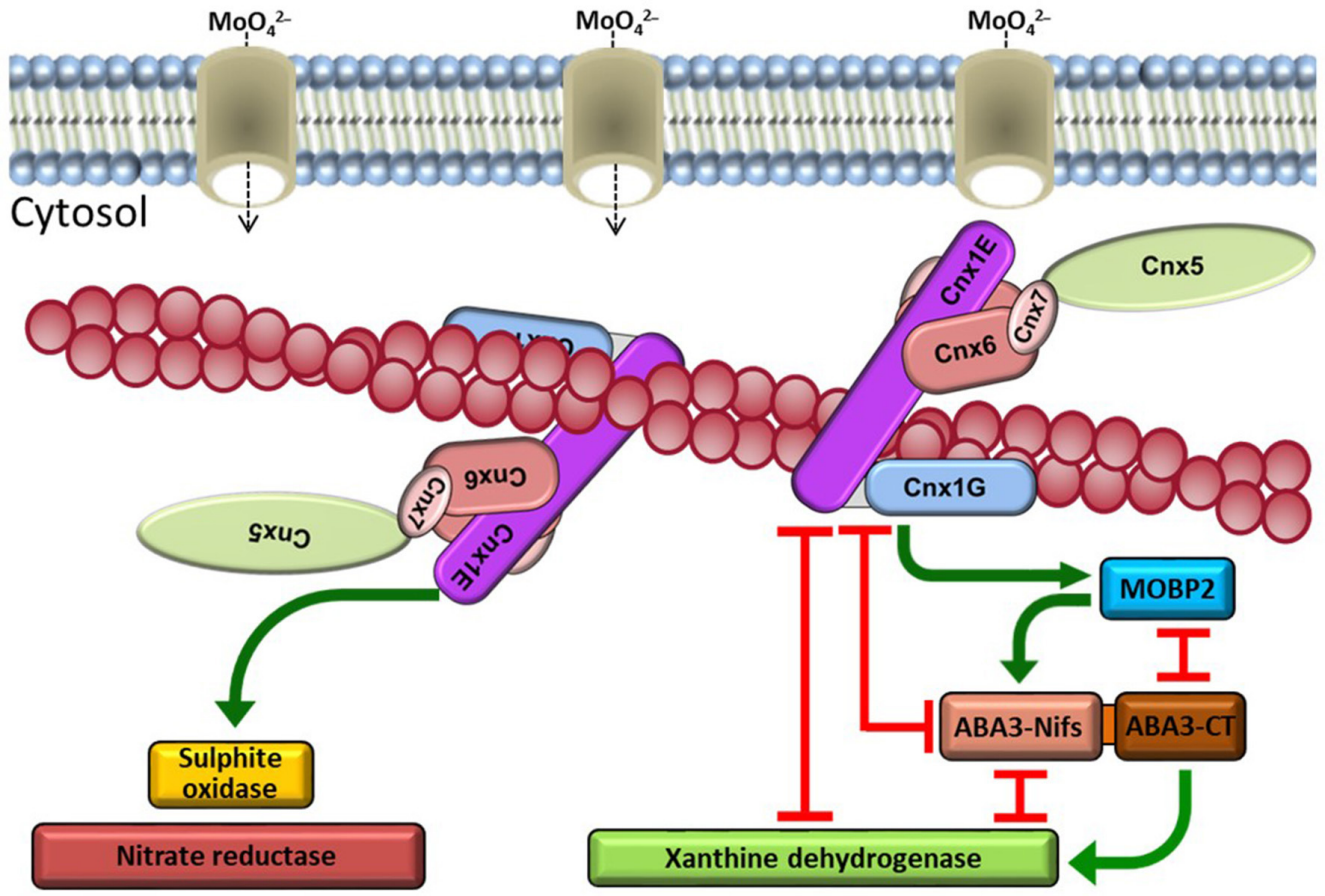
Schematic presentation of the Moco biosynthesis interaction network. The cytosolic Moco biosynthesis enzymes Cnx5, Cnx6, Cnx7, and Cnx1 form a multi-enzyme complex on actin filaments. Molybdate as substrate is provided by molybdate transporters. An assembly of the Moco biosynthesis complex near these transporters at the cytoskeleton is hypothesised. After insertion of Mo from molybdate, di-oxo Moco is inserted into enzymes of the SO family via interaction with Cnx1. The enzymes of the XOR-family receive the mono-oxo form of Moco from ABA3, which generates this form of Moco from di-oxo Moco supplied by Cnx1 via MoBP2 as mediating protein.

For the first time, the complete Moco pathway interaction network has been identified in plants (Figure 2) which might help to understand the closely related biosynthesis in animal and human cells. Although a direct tracking of Moco intermediates through this complex is not practicable with available methods so far, this uncovered protein structure permits the conclusion that micro-compartmentation is a central mechanism inside plant cells to regulate and protect the substrate flow on physiological level.

## Author contributions

DK and RH were primarily involved in drafting the manuscript and DK produced the figures. C-KB and RM critically read the manuscript and improved the text. RRM and RH conceived the study and coordinated the work. All of the authors read and approved the final manuscript.

### Conflict of interest statement

The authors declare that the research was conducted in the absence of any commercial or financial relationships that could be construed as a potential conflict of interest.
